# The application of tissue engineering in cartilage regeneration: technological advances and future challenges

**DOI:** 10.3389/fbioe.2026.1698245

**Published:** 2026-05-25

**Authors:** Hongyu Zhang, Jingwei Feng, Jiajun Zhi, Yiwen Deng, Ronghua Zou, Liulin Fu, Haiyue Jiang

**Affiliations:** 1 Plastic Surgery Hospital, Chinese Academy of Medical Sciences and Peking Union Medical College, Beijing, China; 2 People’s Hospital of Ningxiang City, Changsha, Hunan, China

**Keywords:** biomanufacturing, biomaterial scaffolds, cartilage regeneration, cell-free therapy, clinical translation, regenerative medicine, tissue engineering

## Abstract

Cartilage tissue, owing to its avascular, aneural, and alymphatic nature, possesses a highly limited capacity for self-repair. Damage caused by trauma, degenerative diseases, or congenital malformations seldom heals spontaneously, constituting a long-standing clinical challenge in orthopedic and plastic surgery. Tissue engineering offers a promising strategy for cartilage regeneration by combining seed cells, biomaterial scaffolds, and bioactive molecules to construct substitutes that recapitulate the structure and function of native cartilage. The evolution of biomaterials and scaffold design is traced from natural and synthetic polymers to decellularized extracellular matrix (dECM), nanocomposite scaffolds, and stimuli-responsive hydrogel systems. Advances in biomanufacturing are examined in parallel, with particular attention to the role of 3D/4D bioprinting in fabricating architecturally complex tissue constructs, as well as the contributions of electrospinning and cell sheet engineering. Moving beyond materials and fabrication, the ongoing transition from cell-based regeneration toward cell-free approaches, particularly exosome-mediated endogenous repair and organoid-based micro-tissue construction, is discussed, alongside biomimetic design strategies such as gradient scaffolds, physically responsive scaffolds, and vascular–immune microenvironment regulation. Critical barriers to clinical translation are also identified, including manufacturing costs, process standardization, long-term efficacy and safety validation, patient stratification, and regulatory pathways. The review concludes by outlining future directions such as multi-technology convergence, endogenous regeneration strategies, and the development of off-the-shelf products, with the aim of providing a systematic reference for research and clinical translation in this rapidly evolving field.

## 1 Introduction

Cartilage tissue, characterized by its avascular, aneural, and alymphatic nature, serves essential functions in the human body, facilitating joint movement and maintaining the structural integrity of anatomical features including the auricle, nose, and trachea. However, these very characteristics severely limit its self-repair capacity (Yu L. et al., 2025; Zhang and Yu, 2025). Whether the damage is to articular cartilage due to trauma or degenerative diseases, or a defect in the auricle or nose due to congenital malformation or acquired loss, the tissue is unable to heal effectively. This results in functional impairment and a marked reduction in quality of life, while presenting significant clinical challenges for orthopedic and plastic surgery. Current treatment approaches often yield limited therapeutic outcomes and remain unable to achieve durable, high-quality regeneration of native hyaline cartilage (Kutaish et al., 2025b; Zheng Z. et al., 2025).

**FIGURE 1:**
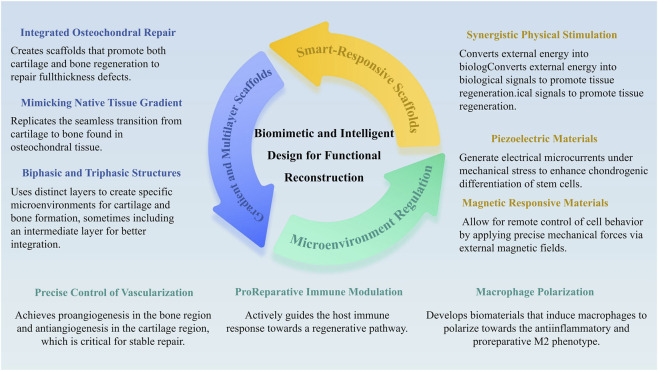
Strategies for biomimetic and intelligent scaffold design.

The emergence of tissue engineering has provided a promising avenue for cartilage regeneration. This field integrates three core components: a suitable cell source (seed cells) to initiate tissue reconstruction, a biomaterial scaffold to provide structural support and guidance for cell growth, and bioactive molecules (such as growth factors) to induce specific cell differentiation and extracellular matrix (ECM) secretion. The ultimate goal is to create engineered substitutes that can mimic the structure and function of native cartilage tissue, thereby guiding and promoting the repair and regeneration of damaged tissue (Wang M. et al., 2024; Wu Z. et al., 2025) (Figure 1). Over the past few decades, considerable advances have been achieved in cartilage regeneration through tissue engineering. Biomaterial design has progressed from simple, inert scaffolds serving merely as structural supports to sophisticated bioactive and stimuli-responsive scaffolds capable of actively modulating cell behavior and recapitulating the native ECM microenvironment. Manufacturing techniques have also advanced from traditional methods like freeze-drying and electrospinning to the era of 3D/4D bioprinting, which allows for the precise construction of complex three-dimensional structures (Loukelis et al., 2024; Zheng Z. et al., 2025). Concurrently, regeneration strategies have expanded from relying on exogenous cell transplantation to using cell-free approaches, such as exosomes and bioactive molecules, and constructing functional microtissues with organoid technology to activate endogenous repair potential (Tian et al., 2024; Hsiung et al., 2025; Tabatabai et al., 2025).

While substantial progress has been made in basic research, clinical translation of cartilage tissue engineering remains fraught with challenges. Key issues that warrant further investigation include the efficient fabrication of biomimetic scaffolds possessing both biological functionality and mechanical stability, the precise regulation of complex regenerative microenvironments encompassing vascularization and immune responses, and the establishment of standardized preclinical evaluation models that satisfy stringent regulatory requirements (Aswathy et al., 2025; Bian et al., 2025; Tor et al., 2025). This review aims to systematically summarize recent technical advances and core challenges in the field of cartilage tissue engineering. It will integrate and analyze the latest cutting-edge literature, focusing on key aspects such as biomaterial and scaffold design, advanced biomanufacturing techniques, biological strategies for driving cartilage regeneration, biomimetic and intelligent designs, and clinical translation. The goal is to provide a theoretical framework and scientific insights for future research directions and clinical applications in this field.

## 2 Biomaterials for cartilage regeneration in tissue engineering: material categories and properties

Biomaterial scaffolds are the core of tissue engineering. They not only provide a three-dimensional physical support for cell adhesion, proliferation, and differentiation but, more importantly, create a microenvironment that mimics the native tissue’s extracellular matrix (ECM) to guide organized tissue regeneration (Vahidi et al., 2024). An ideal scaffold for cartilage regeneration needs to possess excellent biocompatibility, a degradation rate that matches the pace of tissue regeneration, mechanical properties robust enough to withstand joint loads, and biological activity that promotes chondrocyte phenotype maintenance and cartilage matrix synthesis (Wang et al., 2005). In recent years, the deep convergence of materials science and biology has driven the design of cartilage regeneration scaffolds from simple structural mimicry toward functionally integrated and stimuli-responsive composite systems (Lin et al., 2023). The foundational step in this process is the selection of appropriate base biomaterials, whose intrinsic biological and physicochemical characteristics largely determine the regenerative capacity of the resulting scaffold. Accordingly, this part of the review examines three principal categories of base biomaterials—natural polymers, synthetic polymers, and decellularized extracellular matrix with their respective advantages and limitations summarized in Table 1 (Gao et al., 2025).

**TABLE 1:** Comparison of principal biomaterial categories for cartilage tissue engineering.

Biomaterial category	Specific examples	Key advantage	Key disadvantages/Challenges
Natural polymers	Collagen, Silk Fibroin, Chitosan,HA,Alginate	1. Excellent biocompatibility and bioactivity	Animal-derived collagen poses potential risks of immunogenicity and pathogen transmission
		2. Similar structure to natural ECM	
		3. Silk fibroin possesses superior mechanical strength	
Synthetic polymers	PCL,PLGA,PLA	1. Tunable mechanical properties and degradation rates	1. Generally lack bioactivity
		2. Suitable for large-scale production	2. Degradation byproducts (e.g., lactic acid) can create a local acidic environment, which is unfavorable for cell growth
dECM	dECM derived from cartilage, Wharton’s jelly, skin, meniscus, etc	1. Considered the “gold standard” material	Limited source availability, batch-to-batch variation, and potential immunogenicity
		2. A highly biomimetic microenvironment that maximally preserves the native architecture, biochemical composition, and bioactive signals of the ECM	
		3. Effectively guides the chondrogenic differentiation of stem cells and modulates the host immune response	

Abbreviation: dECM, decellularized extracellular matrix; ECM, extracellular matrix; HA, hyaluronic acid; PCL, polycaprolactone; PLGA, Poly (lactic-co-glycolic acid); PLA, polylactic acid.

### 2.1 Natural polymer scaffolds: the application of collagen, silk fibroin, and polysaccharides

Natural polymers have been extensively adopted in cartilage tissue engineering owing to their favorable biocompatibility, intrinsic bioactivity, and structural resemblance to native ECM constituents (Santhamoorthy and Kim, 2025). Collagen, particularly type II collagen, is the main protein component of cartilage ECM and is an ideal choice for creating biomimetic scaffolds (Sun et al., 2025b). Scaffolds based on type I and type II collagen have been shown to effectively support the proliferation and differentiation of chondrocytes and promote the formation of hyaline cartilage (Intini et al., 2025). To overcome the potential immunogenicity and pathogen transmission risks associated with animal-derived collagen, human recombinant collagen produced through genetic engineering offers greater safety and promising applications (Sun et al., 2025b). Silk fibroin derived from silkworms has attracted considerable attention owing to its favorable mechanical strength, tunable degradation rate, and excellent biocompatibility (Tang et al., 2025; Zhu J. et al., 2025). SF-based hydrogels can serve as cell carriers and can also be covalently coupled with growth factors, such as transforming growth factor-β3 (TGF-β3), to present stable biological signals and effectively promote cartilage formation (Majumder et al., 2024). The genetic engineering of SF also provides extensive opportunities for functional design (Ji et al., 2025). Polysaccharide biopolymers, such as chitosan, hyaluronic acid (HA), alginate, and chondroitin sulfate, have unique advantages in mimicking the cartilage microenvironment because their structures are similar to glycosaminoglycans (GAGs) in the cartilage matrix (An et al., 2024; Abraham et al., 2025). Chitosan-based hydrogels have good bioadhesion and antibacterial properties, making them suitable for creating injectable drug delivery systems (Khorasani and Naghib, 2025; Li M. et al., 2025). As a key component of synovial fluid and cartilage ECM, HA derivatives (e.g., methacrylated hyaluronic acid, HAMA) are widely used to develop photocrosslinkable hydrogels and bioinks (Huang P. et al., 2025; Lu et al., 2025). Alginate is often used for cell encapsulation and 3D bioprinting due to its mild gelation conditions (Li C. et al., 2025). Bacterial cellulose, an emerging natural polysaccharide, shows great potential for constructing high-performance composite scaffolds due to its unique nanofiber network structure and high purity (Campano et al., 2025; Yu X. et al., 2025). These natural polymers are frequently used in combination, as exemplified by collagen-HA composite hydrogels and SF-HA scaffolds, to harness their synergistic advantages and more faithfully recapitulate the complex composition and function of native cartilage (Lan et al., 2024; Shi et al., 2024).

### 2.2 Synthetic polymer scaffolds and modification strategies

Synthetic polymers, such as polycaprolactone (PCL), poly (lactic-co-glycolic acid) (PLGA), polylactic acid (PLA), and polyethylene glycol diacrylate (PEGDA), are widely used in cartilage tissue engineering. They offer key advantages, including tunable mechanical properties, controllable degradation rates, and ease of large-scale production (Kamaraj et al., 2025; Nedrelow and Detamore, 2025).

PCL is known for its excellent biocompatibility and mechanical toughness, and it is often used with 3D printing to create scaffolds with specific pore structures for cartilage regeneration (Li H. et al., 2025; Ma et al., 2025). PLGA, a biodegradable polyester, is commonly made into nanoparticles or microspheres to encapsulate growth factors or drugs, enabling controlled local release (Yu X. et al., 2025). However, purely synthetic materials frequently exhibit limited bioactivity, and their degradation byproducts, such as lactic acid, may generate a localized acidic microenvironment that impairs cell proliferation and survival (Wang S. et al., 2025). Therefore, modifying these materials is a crucial strategy to enhance their potential. Common modification methods include blending them with natural polymers, for example, combining PCL with fibrin or PLGA with gelatin to introduce cell recognition sites and improve cell affinity (Ghorbani et al., 2025; Ma et al., 2025). Surface functionalization is another effective strategy. Modifying the surface with cell-adhesion peptides (such as RGD sequences) can significantly enhance cell spreading and proliferation (Akbayrak and Gümüşderelioğlu, 2025). The chemical synthesis of new functional polymers has also become a research hotspot. For instance, incorporating the natural anti-inflammatory molecule xanthohumol into the polyurethane (PEUU) backbone can give the material long-lasting anti-inflammatory activity while also tuning its mechanical properties to better match natural tracheal cartilage (Zheng S. et al., 2025). These modification strategies effectively address the lack of biological function in synthetic materials, giving them a broader application scope in building high-performance cartilage regeneration scaffolds.

### 2.3 Decellularized extracellular matrix scaffolds: mimicking the native microenvironment

Decellularized extracellular matrix (dECM) is widely considered the “gold standard” material for creating tissue engineering scaffolds (Soltanmohammadi et al., 2025). It is produced by removing cellular components from tissues or organs using physical, chemical, or enzymatic methods while preserving the natural 3D structure, biological components, and active signals of the ECM as much as possible. dECM scaffolds provide a highly biomimetic microenvironment rich in collagen, glycosaminoglycans (GAGs), and various endogenous growth factors. These components work together to effectively guide the chondrogenic differentiation of stem cells and modulate the host’s immune response (Gao et al., 2025). dECM can be sourced from a wide variety of tissues, including allogeneic or xenogeneic articular cartilage (Kong et al., 2024), Wharton’s jelly (Gao et al., 2025), skin (Wang H. et al., 2024), meniscus (Huang M. et al., 2025), and even fish (Chen W. et al., 2025) and plant tissues (Hammad et al., 2025), which makes large-scale application possible. dECM can be used in many forms. It can be fabricated into porous solid scaffolds or processed into injectable dECM hydrogels (dECMH) via enzymatic digestion to fill irregularly shaped cartilage defects (Meng et al., 2025). Recently, dECM has also been developed into bioinks for 3D bioprinting, with the goal of precisely replicating the complex anatomical structure of cartilage (Upadhyay et al., 2024; Khalak et al., 2025). Research has shown that dECM scaffolds derived from Wharton’s jelly not only promote the chondrogenic differentiation of bone marrow mesenchymal stem cells (BMSCs) but also inhibit angiogenesis (Gao et al., 2025). This is crucial for maintaining the avascular nature of hyaline cartilage. Furthermore, combining dECM with other biomaterials (like silk fibroin or chitosan) can further optimize the scaffold’s mechanical properties and degradation rate, leading to a synergistic enhancement of both structure and function (Wan et al., 2025). By providing a regenerative microenvironment that is as close to a physiological state as possible, dECM scaffolds offer an extremely promising path toward functional cartilage repair.

## 3 Advanced scaffold design strategies for cartilage regeneration: functional integration and smart-responsive systems

Beyond the intrinsic properties of individual biomaterial categories, the regenerative capacity of a scaffold is critically determined by how the base material is processed, crosslinked, and structurally organized into a functional three-dimensional system. Advanced scaffold engineering involves the integration of sophisticated crosslinking chemistries, composite construction strategies, and nanoscale reinforcement approaches to endow the scaffold with emergent properties (Lin X. et al., 2025)—such as injectability, self-healing capacity, on-demand drug release, and enhanced mechanical toughness—that transcend the capabilities of any single constituent material. By combining multiple biomaterial components into composite systems through rational design, these strategies represent a higher level of structural and functional complexity compared to scaffolds based on a single material category. The following discussion addresses two major scaffold engineering approaches that have attracted considerable attention in recent cartilage tissue engineering research: advanced hydrogel systems that exploit dynamic crosslinking and environmental responsiveness to achieve minimally invasive delivery and intelligent therapeutic regulation, and nanocomposite/hybrid scaffolds that incorporate inorganic or carbon-based nanofillers into polymer matrices to synergistically enhance mechanical performance and biological functionality (Li Z. et al., 2025). The key advantages and limitations of these scaffold design strategies are summarized in Table 2.

**TABLE 2:** Comparison of advanced scaffold design strategies for cartilage tissue engineering.

Scaffold design strategy	Specific examples	Key advantage	Key disadvantages/Challenges
Advanced hydrogels	Injectable hydrogels, self-healing hydrogels, smart-responsive hydrogels	1. Allows for minimally invasive delivery and can fill irregularly shaped defects	Relatively low mechanical strength
		2. Possesses self-healing and tissue-adhesive properties	
		3. Capable of on-demand drug release in response to microenvironmental signals (e.g., MMPs, ROS)	
Nanocomposite/Hybrid scaffolds	Adding nHA, BG, GO, and other nanofillers to the polymer matrix	1. Significantly enhances the mechanical strength, wear resistance, and toughness of the scaffold	Complex fabrication process and the need to ensure uniform dispersion of nanoparticles
		2. Can impart novel biological functions to the scaffold, such as antioxidant properties and magnetic responsiveness	

Abbreviation: BG, bioactive glass; GO, graphene oxide; MMPs, matrix metalloproteinases; nHA, nano-sized hydroxyapatite; ROS, reactive oxygen species.

### 3.1 Advanced hydrogel systems: from injectability to smart responsiveness

Hydrogels are three-dimensional network structures formed by physically or chemically crosslinked hydrophilic polymers. Their high water content, excellent biocompatibility, and mechanical properties similar to natural soft tissues make them one of the most widely used scaffold materials in cartilage tissue engineering (Ansari et al., 2024; Choi et al., 2024; Hashemi-Afzal et al., 2025). Among them, injectable hydrogels have attracted significant attention due to their minimally invasive administration and ability to fill irregular defects (Shaygani et al., 2024; Huang Y. et al., 2025). These hydrogels remain in a sol state under *ex vivo* conditions but undergo rapid *in situ* gelation upon injection, triggered by physiological stimuli including temperature, pH, photoirradiation, or enzymatic catalysis (Yu L. et al., 2025). For example, thermosensitive hydrogels undergo a sol-gel transition at near-physiological temperatures, enabling non-invasive implantation (Yu L. et al., 2025). Beyond injectability, self-healing and tissue-adhesion capabilities are crucial for hydrogels to maintain structural integrity and effectively integrate with surrounding tissues in the dynamic joint environment (Karami et al., 2024; Zhenyuan et al., 2024). Introducing dynamic covalent bonds (such as in Schiff base reactions) or supramolecular interactions can give hydrogels the ability to autonomously repair themselves after damage and enhance their adhesion to cartilage tissue (Zhenyuan et al., 2024). More advanced research focuses on developing “smart” responsive hydrogels. These materials can sense specific signals from the damaged microenvironment, such as elevated matrix metalloproteinases (MMPs) or reactive oxygen species (ROS), and respond by releasing drugs or growth factors on demand (Tong et al., 2024). For instance, one dual-responsive hydrogel system can trigger the release of TGF-β to recruit stem cells via high MMP expression in the joint while simultaneously triggering the release of KGN via mechanical stress from joint movement to promote chondrogenesis. This achieves intelligent spatiotemporal control over the regeneration process (Sun Q. et al., 2025). The development of these advanced hydrogel systems transforms scaffolds from passive structural support into dynamic platforms that can actively participate in and regulate the regeneration process.

### 3.2 Nanocomposite and hybrid scaffolds: enhancing biological function and mechanical properties

To overcome the limitations of single-material scaffolds in terms of mechanical and biological properties, an important trend in cartilage tissue engineering is incorporating nanomaterials into polymer matrices to create nanocomposite and hybrid scaffolds (Elkhoury et al., 2025). Adding nanofillers can significantly improve the scaffold’s mechanical strength, wear resistance, and toughness while also giving it new biological functions. Inorganic nanoparticles like nanohydroxyapatite (nHA) (Zhong et al., 2025), bioactive glass (BG) (Zhu Y. et al., 2025), and layered double hydroxides (LDHs) are widely used for osteochondral repair scaffolds (Luo et al., 2025). These nanoparticles provide mechanical support and can release bioactive ions like calcium, silicon, and strontium as they degrade, synergistically promoting subchondral bone regeneration (Wang et al., 2025c; Zhao et al., 2025). Carbon-based nanomaterials like graphene oxide (GO) and fullerenols also have unique advantages. GO can effectively enhance the mechanical properties and conductivity of hydrogels (Özder et al., 2025), while fullerenols have excellent antioxidant capabilities, which can clear reactive oxygen species (ROS) from the damaged microenvironment and protect chondrocytes from oxidative stress (Liu H. et al., 2025). Additionally, the incorporation of superparamagnetic iron oxide nanoparticles (SPIONs) into hydrogels enables external magnetic fields to modulate the mechanical behavior of the scaffold. SPIONs can also serve as contrast agents for magnetic resonance imaging (MRI), permitting non-invasive monitoring of the repair process (Abtahi et al., 2025). Organic-inorganic hybrid materials based on polyhedral oligomeric silsesquioxane (POSS) offer a new platform for creating advanced scaffolds with controlled mechanical properties and degradation rates, thanks to their unique nanocage structure and numerous modifiable functional groups (Chen M. et al., 2025). By using these nanocomposite and hybrid strategies, researchers are opening up new opportunities for developing next-generation cartilage regeneration scaffolds that have both superior mechanical performance and multiple biological functions.

## 4 Advanced biomanufacturing techniques for cartilage tissue construction

Traditional scaffold fabrication methods, such as solvent casting/particulate leaching and freeze-ing, are easy to perform but have limitations in precisely controlling the scaffold’s internal microstructure (like porosity, pore size, and pore interconnectivity) and macroscopic shape. This makes it difficult to meet the demands for creating complex, biomimetic tissues. In recent years, additive manufacturing techniques, led by 3D/4D bioprinting, have rapidly evolved, bringing about a revolutionary change in cartilage tissue engineering. These techniques make it possible to precisely customize and create structurally complex tissue substitutes based on patient-specific medical imaging data (Jiao et al., 2024). Simultaneously, emerging techniques like electrospinning and cell sheet engineering offer unique and effective ways to construct cartilage tissues with specific microstructures and functions. The core principles, primary benefits, and notable challenges of these key biomanufacturing approaches—namely 3D/4D bioprinting, electrospinning, and cell sheet engineering—are summarized in Table 3.

**TABLE 3:** Comparison of advanced bioprocessing technologies for cartilage tissue engineering.

Technology category	Core principle	Key advantages	Limitations/Challenges
3D/4D bioprinting	Based on computer-aided models, “bioinks” containing cells and bioactive molecules are deposited layer-by-layer to precisely construct 3D tissue architectures. 4D printing incorporates the dimension of “time,” enabling the printed constructs to change in response to stimuli	1. Allows for precise spatial control over the arrangement of materials and cells	The development of ideal bioinks is a key challenge, as they must simultaneously possess both good printability and biological performance
		2. Capable of mimicking the natural stratified structure of cartilage and the gradient characteristics of the osteochondral interface	
		3. Facilitates personalized customization based on patient-specific imaging data	
Electrospinning	Utilizes a high-voltage electrostatic field to stretch a polymer solution or melt into nano- to micro-scale fibers, which are collected to form a non-woven fibrous mat	1. The fabricated fibrous structure effectively mimics the collagen fiber network found in the natural ECM.	Conventional electrospinning technique has limitations in precisely controlling the internal microstructure of the scaffold
		2. Provides excellent topographical guidance for cells	
		3. The derivative technique, MEW, enables the precise and orderly deposition of micro-scale fibers	
CSE	Cells are cultured on smart surfaces, such as thermo-responsive culture dishes. A change in temperature causes the entire cell layer to detach intact, yielding a cell sheet that retains its complete ECM and cell-cell junctions	1. A unique “scaffold-free” tissue engineering approach	The achievable thickness and strength are limited when constructing 3D tissues by stacking multiple cell sheets
		2. Preserves the cell-secreted ECM, exhibiting high bioactivity and strong tissue integration capabilities	
		3. Avoids issues related to scaffold materials, such as cytotoxicity from degradation byproducts and immune responses	

Abbreviation: 3D, three-dimensional; MEW, melt electrowriting; CSE, cell sheet engineering; ECM, extracellular matrix.

### 4.1 3D/4D bioprinting: precision construction of complex tissue structures

3D bioprinting is an additive manufacturing technique that fabricates bioactive three-dimensional tissue constructs through layer-by-layer deposition of bioinks comprising living cells, bioactive factors, and biomaterials, guided by computer-aided design models (Loukelis et al., 2024). The core advantage of this technology is its ability to precisely control the spatial arrangement of materials and cells, allowing it to mimic the layered structure of natural cartilage and the gradient properties of the osteochondral interface (Liu et al., 2024; Heydarigoojani et al., 2025; Khalak et al., 2025). Current 3D bioprinting techniques for cartilage tissue engineering mainly include extrusion-based (Singh et al., 2024), inkjet-based (Khalak et al., 2025), light-assisted techniques, such as stereolithography (SLA) and digital light processing (DLP) (Corrado et al., 2025), and laser-assisted bioprinting (Hall et al., 2024). A crucial step in 3D bioprinting is the development of bioinks. An ideal bioink must have both good printability (e.g., shear-thinning and shape fidelity) and biological performance (e.g., cell compatibility and promotion of chondrogenesis) (Jiao et al., 2024). Researchers have successfully developed various bioinks for cartilage printing, including composite systems based on natural polymers (e.g., alginate-gelatin (Upadhyay et al., 2024), silk fibroin (Akbayrak and Gümüşderelioğlu, 2025), hyaluronic acid (Miklosic et al., 2025)), synthetic polymers (e.g., GelMA (Perry et al., 2025)), and dECM (Kong et al., 2024). Using multi-material printing, it is possible to create layered or gradient scaffolds with different compositions and mechanical properties to mimic the smooth transition from cartilage to bone (Ouyang et al., 2025; Qin et al., 2025).

Building on 3D printing, the concept of 4D bioprinting adds a fourth dimension: time. This technique aims to create “smart” active structures that can change their shape, structure, or function in response to specific physical, chemical, or biological stimuli (Hao et al., 2024; Wu G. et al., 2025). For example, by printing a hydrogel enhanced with magnetic nanoparticles, an external magnetic field can be used to non-invasively apply mechanical stimulation to the implant, thus modulating cell behavior and tissue remodeling (Ioannidis et al., 2025). 4D bioprinting offers an unprecedented ability to mimic the dynamic changes that occur during tissue development and repair, showing immense potential for creating functional cartilage tissue. As illustrated in Supplementary Figure S1, this evolutionary process involves the initial construction of geometries via 3D bioprinting, followed by programmed transformations driven by external physical stimuli to achieve precise adaptation to complex anatomical structures. This shift from static support to dynamic interaction epitomizes the core advantage of 4D fabrication in engineering functional cartilaginous microenvironments.

4D bioprinting endows constructs with the capacity to mimic dynamic changes occurring during tissue development and repair, primarily by leveraging the sensing and feedback capabilities of smart materials toward environmental signals. To further elucidate how these smart-responsive scaffolds drive biological effects through various physical mechanisms, Supplementary Table S1 systematically summarizes multiple signal transduction pathways, including piezoelectric sensing, magneto-thermal effects, and photo-thermal conversion. These mechanisms serve as the theoretical foundation for the functional transformation of 4D-printed structures and provide critical technical support for constructing highly biomimetic and dynamic cartilaginous microenvironments.

### 4.2 Emerging manufacturing techniques: electrospinning and cell sheet engineering

Besides 3D bioprinting, other emerging manufacturing techniques also play a crucial role in cartilage tissue engineering. Electrospinning uses a high-voltage electrostatic field to draw a polymer solution or melt into nano-to micro-scale fibers, which are then collected to form a non-woven fibrous mat. This fibrous structure closely resembles the collagen fiber network in native ECM, providing a favorable topographical guide for cells (Kazmi et al., 2025). By adjusting the spinning parameters, it is possible to precisely control the fiber diameter, orientation, and porosity. Electrospun scaffolds are often used as a reinforcing phase in composite hydrogels to improve their mechanical properties and mimic the anisotropy of cartilage (Cai et al., 2025). Melt Electro-Writing (MEW), a derivative of electrospinning, can deposit micron-sized fibers layer-by-layer with extremely high precision, creating scaffolds with a precise, ordered structure. This offers a unique advantage in mimicking specific cartilage regions, such as the parallel arrangement of collagen fibers in the superficial zone (Snow et al., 2025).

Cell Sheet Engineering (CSE) is a unique scaffold-free tissue construction technique (Chen W. et al., 2024). Cells are cultured on a smart surface, such as a thermoresponsive culture dish, until they form a dense cell monolayer. The cell sheet can then be detached intact by altering the temperature, thereby preserving intercellular junctions and the endogenous ECM secreted by the cells. This ECM-rich cell sheet retains robust biological activity and tissue integration capacity. By stacking multiple cell sheets, three-dimensional tissue constructs with appreciable thickness and mechanical strength can be generated. The mechanical peeling method is a simple and cost-effective way to obtain cell sheets and is widely used in bone and cartilage tissue engineering research (Leisi Mehrabani et al., 2025). The CSE technique avoids problems like the toxicity of degradation products, immune reactions, and mismatched degradation rates that can occur with traditional scaffold materials, providing an innovative solution for creating tissue substitutes composed entirely of cells themselves.

## 5 Biological strategies for driving cartilage regeneration

Successful cartilage regeneration depends not only on a precise scaffold structure and manufacturing technique but also on effective biological strategies to drive cell behavior, including recruitment, proliferation, differentiation, and matrix synthesis. Traditional tissue engineering strategies primarily rely on implanting exogenous cells. Recently, as our understanding of cell-to-cell communication and regenerative microenvironment regulation mechanisms has deepened, emerging biological methods like cell-free therapies and organoid technology are becoming research hotspots. They aim to activate endogenous repair potential in a more refined, physiologically relevant way, leading cartilage regeneration into a new era.

### 5.1 Cell-based regeneration strategies: the application of stem cells and chondrocytes

Cell-based regeneration is a cornerstone of cartilage tissue engineering. The selection of an appropriate cell source is critical, with several distinct cell types commonly utilized, each presenting a unique profile of advantages and disadvantages. Table 4 provides a detailed comparison of the major cell sources for cartilage regeneration, including chondrocytes, Mesenchymal Stem Cells (MSCs), and Induced Pluripotent Stem Cells (iPSCs). Autologous chondrocytes represent a preferred cell source and have demonstrated clinical efficacy in procedures such as autologous chondrocyte implantation (ACI) (Mumme et al., 2025). However, chondrocytes are limited in availability and prone to dedifferentiation during *in vitro* expansion, resulting in loss of the chondrogenic phenotype and production of mechanically inferior fibrocartilage (Diab et al., 2024). To address this, researchers have begun to explore other chondrocyte sources, such as nasal septal and costal chondrocytes, which are not only more abundant but also show a more stable cartilage phenotype and stronger matrix synthesis ability *in vitro* (Chiu et al., 2024; Zheng et al., 2024).

**TABLE 4:** Comparison of major cell sources for cartilage regeneration.

Cell type	Advantages	Disadvantages
Chondrocytes	1. As the primary cell source for autologous cartilage regeneration, they have achieved success in clinical applications such as ACI.	1. Limited source availability
	2. New sources like nasal septum and costal cartilage are more abundant and exhibit a more stable chondrogenic phenotype and stronger matrix synthesis capabilities *in vitro*	2. Prone to dedifferentiation during *in vitro* expansion, leading to the loss of the chondrogenic phenotype and the production of mechanically inferior fibrocartilage
MSCs	1. Considered one of the most promising cell sources due to their multipotent differentiation potential, ease of access, and immunomodulatory properties	​
	2. Can be isolated from various tissues, including bone marrow, adipose tissue, synovial membrane, and umbilical cord	1. Exhibit inherent heterogeneity, which can lead to unstable replication and differentiation efficiency
	3. Low immunogenicity, posing a minimal risk for allogeneic transplantation	2. Face common challenges in all cell-based therapies, including low cell survival rates and potential immune rejection
iPSCs	​	1. Risk of teratoma formation and transplant rejection
	1. Offer a revolutionary solution to cell sourcing, with a nearly unlimited proliferation capacity and the potential to differentiate into all cell types	2. Long-term differentiation protocols are immature, facing challenges of high costs and complex logistics
	2. Can differentiate into chondrocytes to form cartilage-like tissue	3. Also face unstable differentiation efficiency, potential immune rejection, and ethical issues
PBMCs	1. Easy to isolate with minimal invasiveness	The quality of clinical trials and therapeutic efficacy require further investigation to fully harness their potential
	2. Chondrogenic differentiation potential is comparable to that of MSCs	

Abbreviation: PBMCs, Peripheral Blood Mononuclear Cells; iPSCs, Induced Pluripotent Stem Cells; MSCs, Mesenchymal Stem Cells; ACI, autologous chondrocyte implantation.

MSCs are one of the most promising cell sources for cartilage regeneration due to their multipotent differentiation potential, easy accessibility, and immunomodulatory properties (Lin H. et al., 2025). MSCs can be isolated from various tissues, including bone marrow (BMSCs) (Chen et al., 2025c), adipose tissue (ADSCs) (Cho et al., 2025), synovium (SMSCs) (Zheng Y. et al., 2025), umbilical cord, and even urine (Atia et al., 2025). Under specific chemical or physical induction signals, MSCs can differentiate into chondrocytes and secrete cartilage-specific matrix. Combining MSCs with biomimetic scaffolds and growth factors is a major strategy in current cartilage tissue engineering. Furthermore, iPSCs, which are obtained by reprogramming somatic cells, offer a revolutionary solution to the cell source problem because of their nearly infinite proliferation capacity and potential to differentiate into all cell types (Hadzimustafic et al., 2024; Hanaki et al., 2024). While cell-based strategies are promising, they still face challenges such as low cell viability, unstable differentiation efficiency, and potential immune rejection and ethical issues.

### 5.2 Cell-free therapeutic strategies: the rise of exosomes and bioactive molecules

To circumvent the immunogenicity, tumorigenicity, and regulatory complexities inherent in cell-based therapies, cell-free therapeutic strategies have emerged as a rapidly expanding area of investigation (Fan et al., 2024; Wang A. Y. L. et al., 2025). The core of this approach is to directly modulate the microenvironment at the injury site and activate the repair function of endogenous stem cells using bioactive molecules secreted by cells or their carriers.

Exosomes (EXOs) are nanoscale extracellular vesicles (EVs) secreted by cells, which are rich in bioactive substances like proteins, lipids, and nucleic acids (e.g., microRNAs) (Pegtel and Gould, 2019). As key mediators of intercellular communication, exosomes can deliver these “cargoes” to target cells and regulate their physiological functions. Studies have found that exosomes from MSCs have potent pro-regenerative and anti-inflammatory abilities. They can promote chondrocyte proliferation, inhibit apoptosis, and polarize macrophages towards an anti-inflammatory M2 phenotype, creating a microenvironment conducive to cartilage repair (Chen J. et al., 2024; Ye et al., 2025). As illustrated in Supplementary Figure S2, this immunomodulatory process involves the exosome-mediated transition of macrophages from a pro-inflammatory M1 to a pro-reparative M2 profile, thereby suppressing catabolic cytokines and reconstructing the regenerative niche. Loading exosomes into biomaterials like hydrogels allows for sustained release at the defect site, significantly enhancing the therapeutic effect (Villani et al., 2024; Liu L. et al., 2025).

Another important cell-free strategy is the direct application of bioactive molecules. These include various growth factors like the TGF-β family (Caruso et al., 2025; Rodríguez-Cendal et al., 2025), bone morphogenetic proteins (BMPs) (Sun et al., 2025c), and growth differentiation factor 5 (GDF-5) (Zheng Y. et al., 2025), which play a crucial role in cartilage development and homeostasis. Additionally, some small molecules, such as kartogenin (KGN), have been shown to effectively induce the chondrogenic differentiation of stem cells (Bhuyan et al., 2025). However, these bioactive molecules possess short half-lives and are susceptible to rapid inactivation *in vivo*, necessitating efficient delivery systems to preserve their bioactivity and achieve spatiotemporally controlled release. Carriers like nanoparticles (Liang W. et al., 2025), microspheres (Zhang Y. et al., 2025), and smart-responsive hydrogels provide effective solutions for this (Sun Q. et al., 2025). With their higher safety, convenience, and “off-the-shelf” potential, cell-free strategies are opening new pathways for the clinical translation of cartilage regeneration therapies.

To further clarify how these cell-free strategies orchestrate the complex healing process, Supplementary Table S2 summarizes the primary regulatory mechanisms of the regenerative microenvironment, detailed across targets such as regional angiogenesis, immune polarization, and mechanobiological signaling. This multidimensional modulation ensures a “pro-reparative” niche that maximizes the efficacy of bioactive molecule delivery.

### 5.3 Organoid technology: constructing functional cartilage micro-tissues

Organoids are miniature tissues that self-organize from stem or progenitor cells under three-dimensional culture conditions, mimicking some of the structure and function of real organs (Bai et al., 2024; Li et al., 2024). As a bridge between two-dimensional cell culture and *in vivo* models, organoid technology provides a powerful and unprecedented tool for studying organ development, disease modeling, and drug screening. In the field of cartilage tissue engineering, the creation of cartilage organoids (COs) and osteochondral organoids is becoming a Frontier of research (Fu et al., 2025; Wu Y. et al., 2025).

Cartilage organoids are typically formed by culturing MSCs or iPSCs in a specific 3D system (e.g., Matrigel or functionalized hydrogels) under chondrogenic induction (Chen et al., 2025d). These micro-tissues can recreate key events of cartilage development, such as cell aggregation and matrix secretion, forming tissues with structures similar to cartilage lacunae. Osteochondral organoids are of even greater complexity, aiming to recapitulate the entire osteochondral unit comprising the cartilage layer, calcified cartilage layer, and subchondral bone. This enables investigation of the intricate crosstalk between bone and cartilage, which is critical for elucidating the pathological mechanisms underlying diseases such as osteoarthritis (OA) (Smith et al., 2025).

While organoid technology excels at mimicking the tissue microenvironment, the organoids themselves lack a macroscopic structure and sufficient mechanical strength, which limits their direct use for repairing defects in load-bearing joints (Hsiung et al., 2025). To overcome this limitation, researchers have begun to combine organoid technology with biomanufacturing techniques. For example, 3D bioprinting can be used to precisely assemble cartilage organoids as “bio-building blocks” into larger-scale tissue constructs with specific macroscopic shapes and structures, known as “Assembloids” (Dufour et al., 2025; Huang J. et al., 2025). This strategy not only preserves the biological function of the organoids but also gives the constructs macroscopic shape and mechanical stability, representing a significant future direction for organ construction in tissue engineering (Cho et al., 2025).

## 6 Functional reconstruction through biomimetic and intelligent design

To achieve true functional repair of cartilage defects, the design of tissue engineering scaffolds has moved beyond simply carrying cells or filling voids. The focus is now on creating highly biomimetic structures that can replicate the complex architecture, multidimensional functions, and dynamic microenvironment of native tissue. By integrating advanced designs like gradient structures, smart responsiveness, and microenvironment control, new-generation scaffolds aim to actively guide and coordinate the host’s physiological processes to reconstruct long-lasting, functional regenerative tissue.

### 6.1 Gradient and multilayer scaffolds: integrated osteochondral repair

Articular cartilage and the subchondral bone beneath it form a single, interconnected functional unit known as osteochondral tissue. Its structure has a distinct gradient, smoothly transitioning from the superficial hyaline cartilage through a layer of calcified cartilage to the vascularized cancellous bone (Steltzer et al., 2024). Many clinical cartilage injuries involve the subchondral bone, resulting in full-thickness osteochondral defects. Therefore, designing gradient or multilayer scaffolds that can promote both cartilage and bone regeneration is critical for achieving integrated osteochondral repair (Lin X. et al., 2025).

Layered scaffolds, which can be biphasic, triphasic, or even multiphasic, are a common strategy to achieve this goal. Biphasic scaffolds typically have an upper layer that promotes cartilage formation and a lower layer that guides bone formation (Qin et al., 2025). For example, researchers have fabricated a biphasic scaffold via 3D printing, consisting of a collagen upper layer and a GelMA/hydroxyapatite (HAP) lower layer that mimic the microenvironments of cartilage and bone, respectively. This biphasic construct successfully induced lineage-specific differentiation of bone marrow mesenchymal stem cells (BMSCs) (Qin et al., 2025). To better mimic the transition zone of osteochondral tissue, triphasic or multiphasic scaffolds have been developed by including an intermediate layer that mimics calcified cartilage, which can lead to better tissue integration (Lin X. et al., 2025).

However, layered scaffolds can suffer from mechanical mismatch and poor integration at the interfaces between layers. To address this, the concept of continuous gradient scaffolds has emerged. These scaffolds achieve a smooth, continuous change in composition, pore structure, mechanical properties, and bioactive factors within a single structure, more accurately mimicking the seamless transition of native osteochondral tissue (Mahajan et al., 2024; Wang et al., 2025b). Manufacturing techniques like multi-material 3D bioprinting and controlled freeze-drying provide strong support for building these complex gradient structures (Yu et al., 2024). For example, computational methods driven by Design of Experiments (DoE) and artificial intelligence (AI) can optimize printing parameters to precisely control the gradient distribution, creating biomimetic scaffolds that are closer to the structure and function of native tissue (Corrado et al., 2025). These precise biomimetic designs pave the way for reconstructing functional osteochondral interfaces.

To systematically illustrate the complexity of this multi-dimensional design, Supplementary Table S3 compares the differentiated design strategies for the superficial cartilage layer, the middle transition layer, and the subchondral bone base layer across various dimensions, including biomimetic goals, material composition, and fabrication methodologies. Such region-specific precision design serves as a fundamental guarantee for achieving integrated osteochondral regeneration and long-term functional integration.

### 6.2 Smart-responsive scaffolds: physical stimulation for synergistic tissue regeneration

The growth, maintenance, and repair of natural cartilage are intricately regulated by the complex mechanical microenvironment within the joint. Cells sense and respond to these physical stimuli (e.g., pressure, shear force) by adjusting their metabolic activity and matrix synthesis, a process known as mechanotransduction (Huang and Cai, 2025). To mimic this crucial physiological process, researchers are developing “smart” scaffolds that can respond to physical stimuli by converting external energy into beneficial biological signals, thereby synergistically promoting tissue regeneration (Yao et al., 2025).

Piezoelectric biomaterials are particularly suited for this application. Under mechanical stress, including joint loading or external ultrasound stimulation, these materials (e.g., barium titanate nanoparticles) produce surface electrical charges that generate localized microcurrents (Liu Y.-B. et al., 2025). This endogenous electrical stimulation has been shown to significantly promote the chondrogenic differentiation of stem cells and upregulate the expression of cartilage matrix-related genes (Han et al., 2025). Piezoelectric scaffolds made by incorporating piezoelectric nanoparticles into hydrogels can continuously provide electrical stimulation to cells under remote, non-invasive activation by ultrasound, which activates key signaling pathways like Ca/CaM/CaN, thus accelerating cartilage formation (Liu Y.-B. et al., 2025).

Magnetic-responsive materials provide an alternative approach for remote regulation of cell behavior. By embedding magnetic nanoparticles within scaffolds, external static or alternating magnetic fields can exert precise mechanical forces or generate localized heat, thereby simulating physiological mechanical loading (Zhang H. et al., 2025). Combining 4D bioprinting with magnetic materials makes it possible to build dynamic tissue structures that can undergo controlled deformation driven by magnetic fields, providing a new tool for mimicking more complex tissue development processes (Ioannidis et al., 2025). Furthermore, physical therapies like photobiomodulation (PBM) and low-intensity pulsed ultrasound (LIPUS) have also been shown to have a synergistic effect with tissue engineering scaffolds, enhancing the regenerative potential of cells (Zhou et al., 2024; Hang et al., 2025). The design of these smart-responsive scaffolds pushes tissue engineering from static structural replacement to a new phase of dynamic functional regulation.

### 6.3 Microenvironment regulation: the crucial role of vascularization and immune modulation

Tissue regeneration is a complex biological process, and its success largely depends on the microenvironment at the injury site. In osteochondral repair, vascularization and immune response are two critical and often contradictory regulatory targets (Zhou et al., 2025). On one hand, the regeneration of subchondral bone relies on an adequate blood supply, as new blood vessels bring oxygen, nutrients, and osteogenic progenitor cells. On the other hand, a key characteristic of hyaline cartilage is its avascular nature; blood vessel invasion can destroy the cartilage matrix, leading to cartilage calcification and degeneration, which is a major cause of failed cartilage repair (Zhao et al., 2024). Therefore, an ideal osteochondral repair strategy must achieve precise regional control of angiogenesis: pro-angiogenesis in the bone area and anti-angiogenesis in the cartilage area (Liang Z. et al., 2025). Researchers have successfully achieved this by loading different regions of a scaffold with pro-angiogenic factors (like VEGF) or anti-angiogenic factors (like Axitinib), or by using biomaterials with natural anti-angiogenic properties (such as dECM from Wharton’s jelly). This promotes the regeneration of a stable osteochondral interface (Liang Z. et al., 2025; Zhou et al., 2025).

Implanted biomaterials inevitably interact with the host’s immune system, triggering an immune response that directly affects the success of material integration and tissue repair (Maduka et al., 2024). While conventional perspectives emphasized suppressing the immune response, the contemporary concept of immunoengineering seeks to actively redirect immune activity toward a pro-regenerative phenotype (Nie et al., 2024). Macrophages are key regulators in this process; they can polarize into either a pro-inflammatory M1 phenotype or an anti-inflammatory, pro-reparative M2 phenotype. Many studies focus on developing biomaterials that can induce macrophage polarization towards the M2 phenotype, either by tuning the material’s physicochemical properties or by loading immunomodulatory factors (such as certain cytokines or exosomes) (Ye et al., 2025). By actively regulating the vascular and immune microenvironment, a “pro-reparative” niche can be created for tissue regeneration, significantly increasing the success rate of tissue engineering strategies.

## 7 Evaluation and clinical translation of cartilage regeneration technologies

While significant progress has been made in basic research for cartilage tissue engineering, successfully translating these technologies “from bench to bedside” remains a major challenge. This process requires rigorous preclinical evaluation to confirm a new therapy’s safety and efficacy, as well as navigating complex clinical translation pathways and strict regulatory requirements.

### 7.1 Progress in preclinical evaluation models and methodologies

Preclinical evaluation is the critical bridge connecting basic research and clinical trials. Its goal is to systematically assess the efficacy and safety of new therapeutic strategies under controlled experimental conditions. Animal models are essential for this process. Depending on the research objective, a range of animal models can be used, from small rodents (e.g., rats, rabbits) to larger animals (e.g., goats, pigs, dogs) that are closer to humans in joint size and load (Long et al., 2024; Meng et al., 2024). Results from large animal models are more indicative for clinical translation because their anatomical structure, physiological load, and cartilage repair responses more closely resemble those of humans.

The selection of an appropriate animal model is crucial for translational research, as each species offers distinct advantages and limitations. Rabbits are commonly used for proof-of-concept and biocompatibility studies due to their low cost, ease of surgical handling, and well-characterized biology (Chu et al., 2010). However, rabbits have thin articular cartilage, small joint dimensions, and a higher intrinsic healing capacity, which may lead to overestimation of therapeutic efficacy and limit their translational predictive value. For translational studies supporting regulatory approval and clinical application, large animal models are essential, especially goats and horses. Goats are widely employed for osteochondral defect repair due to their moderate joint size, cartilage thickness (0.8–2.0 mm), and similarity of the caprine stifle to the human knee joint, though differences in stifle joint flexion angles between goats and humans should be considered (Moran et al., 2016). The equine model is recognized as the most clinically relevant large animal model for articular cartilage repair, with cartilage thickness (2.0–3.0 mm) most closely approximating that of the human knee. Furthermore, equine articular cartilage exhibits a similar low intrinsic repair capacity as human cartilage, making it highly relevant for studying cartilage repair (McIlwraith et al., 2011; Moran et al., 2016). Despite its advantages, the equine model has significant practical constraints, including high procurement and housing costs, specialized veterinary surgical facilities, and substantial ethical considerations. Therefore, a tiered approach to preclinical evaluation is recommended: small animal models (primarily rabbits) for initial proof-of-concept and mechanism studies, intermediate large animal models (goats or minipigs) for efficacy optimization, and equine models for pivotal translational studies most closely predicting clinical outcomes in humans (Chu et al., 2010; McIlwraith et al., 2011; Moran et al., 2016).

In recent years, to reduce reliance on animal testing and improve research efficiency, *in vitro* and *ex vivo* models have rapidly advanced. *In vitro* models based on organoids and “organ-on-a-chip” technology can simulate the complex microenvironment and pathological processes of cartilage tissue, providing an efficient platform for drug screening and mechanistic studies (Dönges et al., 2024). *Ex vivo* models use fresh cartilage tissue blocks from animal or human surgical specimens to simulate the injury and repair process outside the body, offering a valuable tool for assessing the interaction between biomaterials and tissue (Trengove et al., 2024). Additionally, innovative tracking techniques, such as using dual-fluorescent-labeled hydrogels to monitor material degradation and tissue formation in real time, provide new perspectives for a deeper understanding of regeneration mechanisms (Li S. et al., 2025).

### 7.2 Clinical translation: current status, challenges, and regulatory science

The overarching goal of cartilage tissue engineering is the development of safe and efficacious therapeutic products suitable for routine clinical use. At present, ACI and its modified variants represent the limited number of cell-based therapies that have achieved broad clinical adoption (Kutaish et al., 2025b). However, ACI has drawbacks such as extensive surgical trauma, high costs, and the need for a second surgery (Mumme et al., 2025). Recently, several next-generation tissue engineering products have entered clinical trials, showing promising potential. For example, a multicenter randomized controlled clinical trial demonstrated that an engineered cartilage graft (N-TEC) constructed with autologous nasal septal chondrocytes was clinically more effective than traditional matrix-assisted chondrocyte implantation for repairing knee cartilage defects, especially for large-area defects and revision cases, validating the clinical significance of *in vitro* tissue maturation (Mumme et al., 2025). Furthermore, “off-the-shelf” engineered cartilage microspheres based on allogeneic donor chondrocytes have shown safety and efficacy in large animal models and have entered Phase I/IIa clinical trials (Kutaish et al., 2025a).

Despite this promising outlook, the clinical translation of cartilage tissue engineering still faces many significant challenges. First, there are production and cost issues. The process of cell sourcing, *in vitro* expansion, and tissue construction is complex and requires expensive facilities and strict quality control to meet good manufacturing practice (GMP) standards, leading to high treatment costs that limit widespread use (Tor et al., 2025). Second, there is a lack of standardization and reproducibility. From cell sources and scaffold fabrication to surgical procedures, there is no uniform standard for each step, leading to high heterogeneity in clinical study results and making direct comparison and evaluation difficult (Corrado et al., 2025).

An equally critical challenge that has emerged from the clinical experience to date is the insufficient integration of personalized medicine principles into cartilage regeneration strategies. Cartilage defects are not a monolithic clinical entity; they vary substantially in etiology, size, depth, anatomical location, and the biological condition of the surrounding tissue. Patients themselves present with considerable heterogeneity in age, body mass index, activity level, comorbidities, systemic inflammatory burden, and the degenerative stage of the affected joint. Personalized medicine, in this context, refers to the systematic tailoring of therapeutic approaches to the individual patient’s specific clinical, biomechanical, and molecular profile, with the goal of maximizing treatment efficacy while minimizing unnecessary risk and cost.

The role of personalized medicine in cartilage therapeutic strategies operates at multiple levels. At the treatment selection level, it involves matching the optimal therapeutic modality to the patient’s condition. For example, young patients with focal traumatic chondral defects in otherwise healthy joints may be best suited for cell-based approaches such as autologous chondrocyte implantation or scaffold-assisted repair, whereas older patients with diffuse degenerative changes and an inflammatory joint microenvironment may derive greater benefit from cell-free therapies, such as exosome-laden hydrogels or bioactive molecule delivery systems designed to modulate the local immune response (Fan et al., 2024; Ye et al., 2025). At the implant design level, patient-specific medical imaging data can be leveraged to fabricate anatomically customized scaffolds through 3D/4D bioprinting, ensuring precise geometric fit and appropriate mechanical loading characteristics for each individual defect ((Loukelis et al., 2024; Corrado et al., 2025). At the biological level, the use of autologous or patient-matched cell sources—including iPSC-derived chondrocytes or autologous MSC-derived exosomes—represents an inherently personalized strategy aimed at reducing immunogenicity and improving graft integration (Hadzimustafic et al., 2024; Wang A. Y. L. et al., 2025).

Despite the clear rationale for such individualized approaches, the majority of clinical trials conducted in cartilage repair have historically adopted broad and poorly stratified patient inclusion criteria, enrolling heterogeneous populations without systematic consideration of the underlying phenotypic condition. In the context of osteoarthritis-associated cartilage damage, distinct clinical phenotypes—including inflammatory, mechanical load-driven, chronic pain, metabolic syndrome, and bone-cartilage metabolism subtypes—have been increasingly recognized through systematic analyses, each governed by different pathological mechanisms and, critically, likely to respond differently to different therapeutic interventions (Dell’Isola et al., 2016). More importantly, the heterogeneous nature of osteoarthritis has been directly implicated in the failure of multiple phase 3 clinical trials for disease-modifying osteoarthritis drugs, where promising preclinical and early clinical results failed to replicate in broadly recruited patient populations (Van Spil et al., 2019). When phenotypically diverse patients are grouped together without stratification, genuine therapeutic effects within responsive subpopulations may be diluted and obscured, leading to the erroneous conclusion that a given intervention lacks efficacy.

To address this, the future clinical translation of cartilage tissue engineering must incorporate robust patient phenotyping and stratification strategies at the trial design stage. This requires the development and clinical validation of reliable biomarkers capable of classifying patients prior to treatment. Candidate biomarkers include inflammatory mediators and catabolic enzymes in synovial fluid, quantitative MRI parameters such as T2 mapping and delayed gadolinium-enhanced MRI of cartilage (dGEMRIC) that reflect cartilage composition and integrity, and emerging molecular signatures derived from proteomic or transcriptomic profiling of joint tissues (Fw et al., 2022). Furthermore, the integration of artificial intelligence with multi-omics data holds promise for refining predictive models of individual treatment response. Adopting adaptive or biomarker-stratified clinical trial designs, in which patient enrollment is enriched for subgroups most likely to benefit from a specific intervention, will be essential to demonstrate true therapeutic efficacy, reduce trial failure rates, and ultimately accelerate regulatory approval of next-generation cartilage regeneration therapies.

Additionally, long-term efficacy and safety still need to be confirmed with more high-quality clinical evidence. It is crucial to address whether regenerated cartilage can maintain its structure and function long-term in the complex mechanical and biochemical environment of a joint, as well as the long-term safety of stem cell applications (e.g., tumorigenic risk) (Athiviraham, 2025).

**FIGURE 2:**
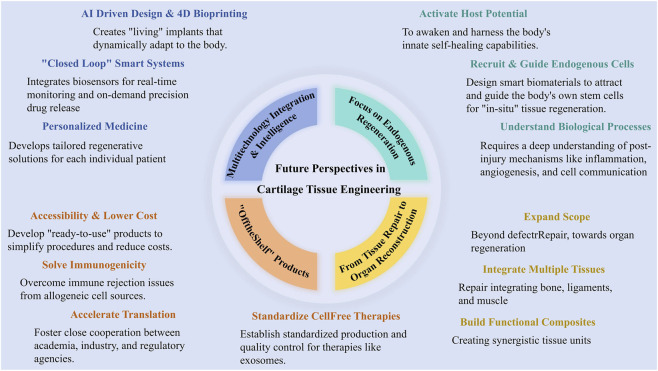
Future perspectives of cartilage tissue engineering.

Finally, regulatory science is the essential path to clinical application. As a new class of “living” drugs or medical devices, tissue engineering products have complex approval pathways with varying regulations across countries. These products are typically classified as Advanced Therapy Medicinal Products (ATMPs), which must meet strict regulatory requirements. Therefore, researchers, companies, and regulatory agencies need to collaborate more closely to establish clear, scientific review standards and translation pathways to accelerate the availability of safe and effective new technologies to patients (Figure 2).

## 8 Conclusion and future perspectives

### 8.1 Conclusion

Cartilage tissue engineering as one of the most challenging and rapidly evolving branches of regenerative medicine, cartilage tissue engineering has witnessed substantial progress over the past several decades. Spanning from fundamental biomaterials research to advanced biomanufacturing, and from single-cell therapies to sophisticated microenvironment regulation, the field has continuously expanded in scope and depth, providing new avenues for addressing the longstanding clinical challenge of cartilage repair. A retrospective analysis of current advances reveals a distinct technological trajectory, shifting from mere structural replacement to functional reconstruction. Early research focused on developing scaffolds with good biocompatibility and mechanical support to provide a basic growth space for cells. Today, the research focus has shifted to creating “smart” and “living” implants that can actively guide tissue regeneration and dynamically interact with the host environment through highly biomimetic design.

Currently, advanced hydrogel systems, dECM-based matrices, 3D/4D bioprinting technologies, cell-free regeneration strategies (especially exosomes and organoid technology), and multi-functional gradient scaffold design collectively form the core driving force of modern cartilage tissue engineering. The chemical and physical properties of hydrogels can be finely tuned to give them injectability, self-healing, tissue adhesion, and smart responsiveness to the microenvironment. dECM materials preserve the biological information of native tissue to the greatest extent possible, providing an optimal regenerative “soil” for cells. 3D/4D bioprinting integrates these advanced materials with cells and bioactive factors with high precision, enabling personalized customization of a tissue’s macroscopic shape and microscopic structure. The rise of cell-free therapies and organoid technologies not only offers new ways to bypass the inherent risks of cell therapy but also opens new windows for us to deeply understand cartilage development and disease mechanisms.

### 8.2 Outlook

Looking ahead, the development of cartilage tissue engineering will follow several key trends.

#### 8.2.1 Multi-technology integration and intelligence

Future research will focus more on the cross-disciplinary integration of technologies. For example, combining AI-driven algorithm design with high-precision 4D bioprinting will enable the creation of “living” implants that can mimic the dynamic changes of cartilage throughout its life cycle (Wei et al., 2025). Developing “closed-loop” smart repair systems that integrate biosensors and responsive materials to monitor repair status in real-time and release therapeutic drugs on demand will be an important step towards personalized medicine.

#### 8.2.2 Focus on endogenous regeneration

Harnessing the host’s inherent repair capacity via endogenous regeneration constitutes a central objective of next-generation regenerative medicine. Future efforts will place greater emphasis on the design of biomaterials that precisely modulate the local microenvironment, recruiting and guiding endogenous stem/progenitor cells while finely regulating their cell fate to realize “*in situ*” tissue regeneration (Liu Y.-B. et al., 2025). This will require a deeper understanding of complex biological processes following cartilage injury, such as inflammatory responses, angiogenesis, and cell-to-cell communication.

#### 8.2.3 Development and clinical translation of “off-the-shelf” products

To reduce costs, simplify treatment procedures, and increase accessibility, the development of “off-the-shelf” tissue engineering products is an inevitable trend. This depends on solving the immunogenicity issues of allogeneic cell sources (like iPSCs and allogeneic chondrocytes) and standardizing the production and quality control of cell-free therapies (like exosome preparations) (Kutaish et al., 2025a). At the same time, accelerating clinical translation requires close cooperation among academia, industry, and regulatory agencies to establish clearer, more efficient evaluation standards and approval pathways, along with more rigorously designed, high-quality clinical studies with long-term follow-up (Mumme et al., 2025).

#### 8.2.4 From tissue repair to organ reconstruction

As technology matures, the scope of cartilage tissue engineering will expand from repairing focal defects to the bioengineering reconstruction of entire joints and even more complex organs (such as the trachea and ears) (Song et al., 2024; Lin W. et al., 2025). This will require not only the ability to reconstruct cartilage but also to integrate multiple tissues like bone, ligaments, synovium, and muscle to build functional multi-tissue composites.

In conclusion, cartilage tissue engineering stands at a critical juncture where technological advancement is accelerating and its translational potential is becoming increasingly apparent. While the road ahead is still full of challenges, with our deepening understanding of cartilage biology and materials science and the continuous emergence of innovative technologies, there is reason to believe that functional and durable cartilage regeneration will transform from a scientific vision into a clinical reality in the near future, bringing hope to millions of patients with joint diseases worldwide.

## Supplementary material

SUPPLEMENTARY FIGURE S1 Schematic of 4D bioprinting and stimuli-responsive transformation for functional cartilage reconstruction. This figure illustrates the evolution from static 3D printing to dynamic 4D biofabrication (I) 3D Bioprinting and Crosslinking: Bioinks containing chondrocytes and functional nanoparticles (e.g., magnetic or piezoelectric) are deposited layer-by-layer to form an initial programmed geometry (II) External Stimulus Induction: The printed construct is exposed to targeted external stimuli such as near-infrared (NIR) light, magnetic fields, or temperature changes (III) 4D Shape Transformation: The scaffold undergoes a programmed shape transformation over time, allowing it to dynamically transition from a flat structure to an anatomically matched 3D substitute (e.g., a meniscus or ear construct) that fits complex tissue defects.

SUPPLEMENTARY FIGURE S2 Mechanism of cell-free exosome therapy in modulating the cartilage regenerative microenvironment. Mechanistic illustration of how cell-free scaffolds reshape the injury niche. Baseline State: Following injury, the microenvironment is dominated by pro-inflammatory M1-like macrophages that release catabolic cytokines (e.g., TNF-α, IL-1β), leading to persistent cartilage degradation. Advanced Regenerative State: A biomimetic scaffold loaded with exosomes provides sustained release of bioactive cargoes. These exosomes trigger a phenotype switch (polarization) from M1 to anti-inflammatory M2-like macrophages. This shift suppresses abnormal angiogenesis in the cartilage layer while promoting a “pro-reparative” niche characterized by anti-inflammatory signaling and endogenous tissue regeneration.
