# Supplementary material for: The application of tissue engineering in cartilage regeneration: technological advances and future challenges
Source: Front Bioeng Biotechnol. 2026 May 25;14:1698245. doi: 10.3389/fbioe.2026.1698245 (PMC13243408; doi:10.3389/fbioe.2026.1698245)
Supplement: Supplementary file 2 [file Image1.PDF]

## *Supplementary Figures*

# *Contents*

***Supplementary Figure 1*** .......... 2

***Supplementary Figure 2*** .......... 3

*Supplementary Figure 1*

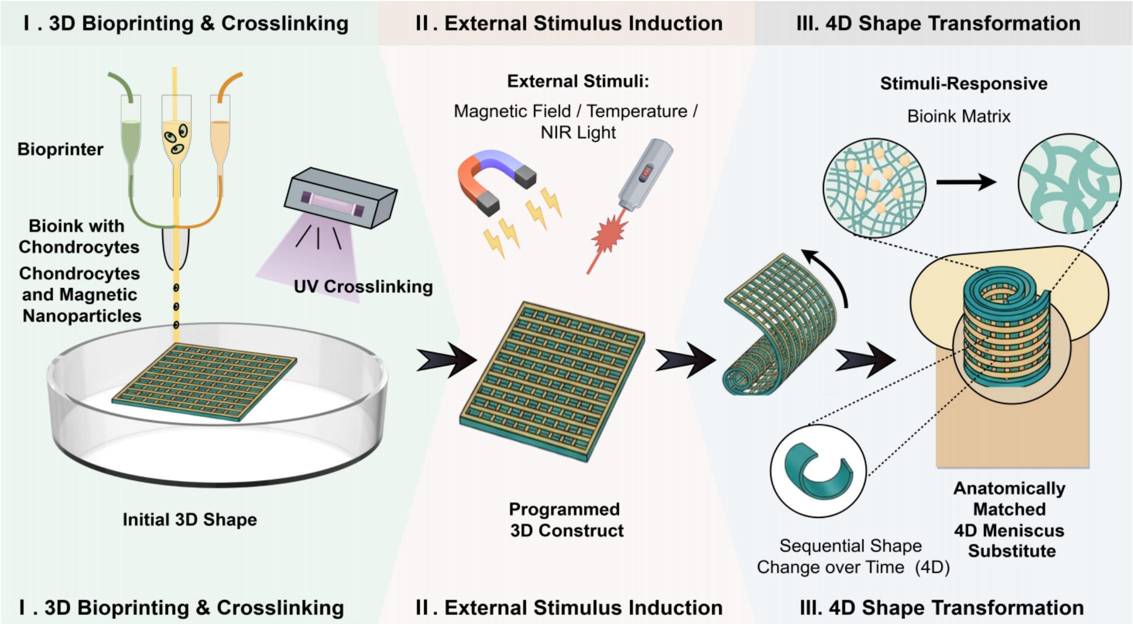


**Supplementary Figure 1.** Schematic of 4D bioprinting and stimuli-responsive transformation for functional cartilage reconstruction.

This figure illustrates the evolution from static 3D printing to dynamic 4D biofabrication. (I) 3D Bioprinting & Crosslinking: Bioinks containing chondrocytes and functional nanoparticles (e.g., magnetic or piezoelectric) are deposited layer-by-layer to form an initial programmed geometry. (II) External Stimulus Induction: The printed construct is exposed to targeted external stimuli such as near-infrared (NIR) light, magnetic fields, or temperature changes. (III) 4D Shape Transformation: The scaffold undergoes a programmed shape transformation over time, allowing it to dynamically transition from a flat structure to an anatomically matched 3D substitute (e.g., a meniscus or ear construct) that fits complex tissue defects.

***Supplementary Figure 2***

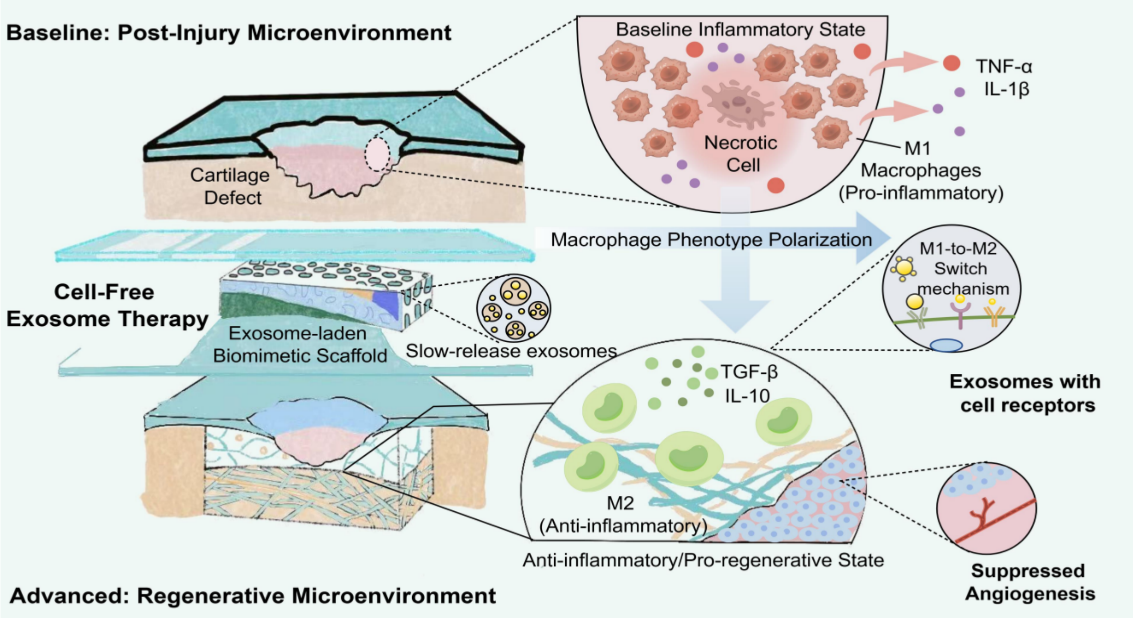


**Supplementary Figure 2.** Mechanism of cell-free exosome therapy in modulating the cartilage regenerative microenvironment.

Mechanistic illustration of how cell-free scaffolds reshape the injury niche. Baseline State: Following injury, the microenvironment is dominated by pro-inflammatory M1-like macrophages that release catabolic cytokines (e.g., TNF-α, IL-1β), leading to persistent cartilage degradation. Advanced Regenerative State: A biomimetic scaffold loaded with exosomes provides sustained release of bioactive cargoes. These exosomes trigger a phenotype switch (polarization) from M1 to anti-inflammatory M2-like macrophages. This shift suppresses abnormal angiogenesis in the cartilage layer while promoting a "pro-reparative" niche characterized by anti-inflammatory signaling and endogenous tissue regeneration.
